# IFN-Dependent and -Independent Reduction in West Nile Virus Infectivity in Human Dermal Fibroblasts

**DOI:** 10.3390/v6031424

**Published:** 2014-03-24

**Authors:** Lisa I. Hoover, Brenda L. Fredericksen

**Affiliations:** 1 Maryland Pathogen Research Institute, College Park, MD 20742, USA; E-Mail: linjaian@umd.edu; 2 Department of Cell Biology and Molecular Genetics University of Maryland, College Park, MD 20742, USA

**Keywords:** West Nile virus, interferon, human dermal fibroblast, antiviral response, intracellular innate immunity

## Abstract

Although dermal fibroblasts are one of the first cell types exposed to West Nile virus (WNV) during a blood meal by an infected mosquito, little is known about WNV replication within this cell type. Here, we demonstrate that neuroinvasive, WNV-New York (WNV-NY), and nonneuroinvasive, WNV-Australia (WNV-AUS60) strains are able to infect and replicate in primary human dermal fibroblasts (HDFs). However, WNV-AUS60 replication and spread within HDFs was reduced compared to that of WNV-NY due to an interferon (IFN)-independent reduction in viral infectivity early in infection. Additionally, replication of both strains was constrained late in infection by an IFN-β-dependent reduction in particle infectivity. Overall, our data indicates that human dermal fibroblasts are capable of supporting WNV replication; however, the low infectivity of particles produced from HDFs late in infection suggests that this cell type likely plays a limited role as a viral reservoir *in vivo*.

## 1. Introduction

West Nile virus (WNV) is a neurotropic *Flavivirus* that has recently emerged as a significant threat to human health. Prior to the 1990s, most WNV infections were asymptomatic or associated with a mild febrile illness known as West Nile fever. However, the recent introduction of WNV into naïve populations in Europe, Israel, and the Americas has resulted in a marked increase in both the number of reported cases and the severity of disease when compared to previous outbreaks. WNV is now the leading cause of mosquito-borne neuroinvasive disease in the United States. Between 1999 and 2013, over 17,000 cases with neurological complications, such as meningitis, encephalitis, and acute flaccid paralysis, and over 1600 deaths due to WNV were reported [1]. The increased virulence of recently emerged strains of WNV represents a significant public health concern since antiviral therapies and vaccines are not currently available for use in humans.

The WNV genome is approximately 11 kb in length and consists of a single open reading frame encoding three structural proteins (Capsid, Premembrane, and Envelope) and seven nonstructural proteins (NS1, NS2A, NS2B, NS3, NS4A, NS4B, and NS5). Based on the phylogenetic analysis of partial genomic sequences of structural genes, WNV has been grouped into five lineages and two clades, which differ from each other by 20%–27% [2,3]. Most strains reside within the two main lineages, designated Lineage 1 and Lineage 2. Lineage 1 strains have been isolated from North America, Europe, the Middle East, Africa, Asia and Australia. While Lineage 2 strains were initially confined to sub-Saharan Africa, they have recently been detected in eastern and southern Europe as well as South Africa [4,5,6,7,8,9,10,11,12]. Experimental infections in rodents and birds demonstrated that the virulence and neuroinvasiveness of strains from both Lineage 1 and 2 are highly variable, ranging from nonpathogenic to highly neuroinvasive [13,14,15,16,17].

WNV is primarily maintained in nature in an enzootic transmission cycle between avian hosts and mosquito vectors. Though mosquitos can transmit WNV to humans and other mammals, this normally results in a dead-end infection since levels of viremia are not sufficient for transmission back to the mosquito vector [18]. Transmission of WNV to avian and mammalian hosts occurs when an infected mosquito deposits saliva containing high doses of virus into the dermal layer of the skin while probing for a blood vessel [19,20,21,22,23]. The deposited virus is thought to infect local skin cells as well as immune cells that are recruited to the inoculation site, such as neutrophils and Langerhans dendritic cells [24]. Studies with WNV and dengue virus suggest that the immune cells promote viral dissemination by transporting the virus to draining lymph nodes, where a second round of replication occurs [25,26,27,28,29]. The amplified virus then enters the circulatory system via the efferent lymphatic system and the thoracic duct. The subsequent viremia allows WNV to access distal organs, including the spleen, heart, liver, kidneys, and brain. 

As one of the first cell types exposed to WNV during a mosquito’s blood meal, the nonmigrating cells within the skin may function as an early reservoir for WNV infection. Keratinocytes, the major cell type comprising the epidermal layer of the skin, have been shown to support high levels of WNV replication [30]. Moreover, primary human dermal fibroblasts are also capable of sustaining the replication of WNV as well as several other *Flaviviruses* [31]. However, little else is known about WNV replication within cells comprising the dermal layer of the skin. Therefore, we further assessed the ability of WNV to propagate in primary human dermal fibroblasts. Specifically, we compared the ability of a neuroinvasive, WNV-New York (WNV-NY) [32], and a nonneuroinvasive, WNV-Australia (WNV-AUS60) [16], Lineage I strain to replicate in these cells. While both strains of WNV replicated in human dermal fibroblasts, WNV-AUS60 achieved lower overall peak viral titers compared to WNV-NY. Furthermore, IFN-β affected the replication and particle infectivity of both strains at late, but not early, times post-infection. Although neutralization of IFN-β increased particle infectivity for both strains late in infection, a significant difference in WNV-AUS60 and WNV-NY particle infectivity remained.

## 2. Results and Discussion

### 2.1. WNV Replication in Human Dermal Fibroblasts

**Figure 1:**
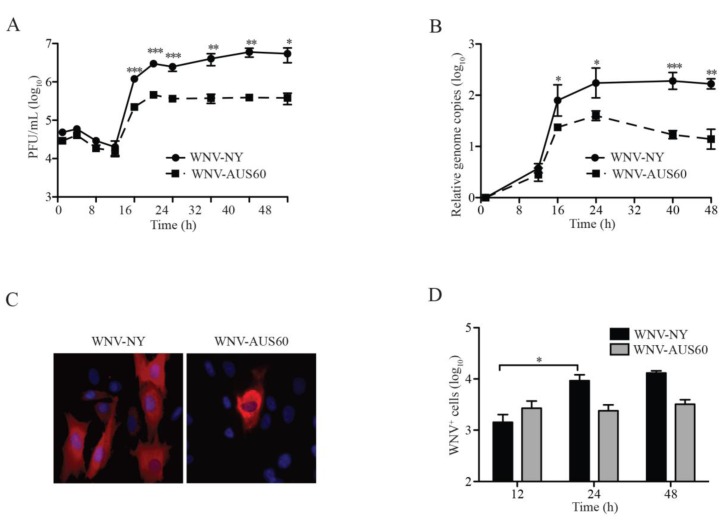
West Nile virus (WNV) replication in human dermal fibroblasts (HDFs). HDF cells were infected (MOI = 0.05) with WNV-NY or WNV-AUS60. (**A**) WNV infectious particles production in HDFs. Culture supernatants were recovered at the indicated times and titered by plaque assay on Vero cells. Values represent the average number of plaque forming units (PFU) per mL (±standard error) from three independent experiments; (**B**) RNA synthesis of WNV-NY and WNV-AUS60 in HDFs. Total RNA was extracted from cells at the indicated times and viral RNA levels were assessed by qRT-PCR. Relative WNV genome copies were calculated as a change in WNV genome copies per ng of RNA from 1 h post-infection. Values represent the average (±standard error) of at least three independent experiments; (**C**) Examination of viral protein expression by immunofluorescence assay (IFA). HDFs were fixed with 3% paraformaldehyde at 24 h post-infection, permeabilized, dyed with Hoescht stain (blue), and probed with WNV hyperimmune ascitic fluid and goat anti-mouse IgG 549 nm-Dylight conjugated secondary antibody (red). Images are representative of at least three independent experiments; (**D**) WNV spread in HDF. The number of infected cells within the monolayer was determined by flow cytometry. Monolayers were trypsinized at the indicated times, fixed with 3% PFA and probed with WNV hyperimmnue serum. Values represent the average number (±standard error) of WNV-positive cells per 10^5^ cells from three independent experiments. Statistical significance was determined by an unpaired t-test. Asterisks indicate differences that are statistically significant (* *p* < 0.05; ** *p* < 0.01; *** *p* < 0.001).

Because dermal fibroblasts are likely one of the first cell types to become infected following the blood meal of a mosquito, we compared the replication kinetics of insect cell-passaged WNV-NY and WNV-AUS60 in human dermal fibroblasts (HDFs) using a multistep growth curve. Both neuroinvasive, WNV-NY, and nonneuroinvasive, WNV-AUS60, strains replicated within HDFs without obvious induction of cytopathic effects (CPE) (data not shown) and reached peak infectious particle production between 20 and 24 h post-infection (Figure 1A). However, peak viral titers of WNV-AUS60 were approximately one log lower than WNV-NY. Additionally, quantitative RT-PCR (qRT-PCR) analysis revealed that the kinetics of WNV-NY and WNV-AUS60 genome replication occurred at similar rates (Figure 1B). Peak levels of viral genome accumulation were detected at 24 h post-infection and lower levels of viral genomic RNA were detected at all times in WNV-AUS60-infected cells compared to WNV-NY-infected cells. Therefore, although both strains were capable of establishing an infection within HDFs, the nonneuroinvasive strain, WNV-AUS60, never reached levels of replication as high as the neuroinvasive strain, WNV-NY. 

We hypothesized that WNV-AUS60 multiplication in HDFs is reduced compared to that of WNV-NY, due to a defect in cell-to-cell spread. Therefore, we examined viral protein expression in infected cells by immunofluorescence assay (IFA). While multi-cell foci of infected cells were detected in WNV-NY-infected cultures after 24 h, WNV protein expression was primarily restricted to single cells within WNV-AUS60-infected cultures. The detection of unicellular foci suggests that WNV-AUS60 is restricted in its ability to spread from cell to cell within the HDF monolayer compared to WNV-NY (Figure 1C). To confirm this result, we quantitated the number of infected HDFs over the course of infection using flow cytometry (Figure 1D). Similar levels of WNV-positive cells were detected at 12 h post-infection in WNV-NY- and WNV-AUS60-infected cultures, indicating that both viruses initially established comparable levels of infection within the HDF monolayer. However, the number of WNV-NY-positive cells increased between 12 and 24 h post-infection, whereas the number of WNV-AUS60-positive cells remained unchanged. Thus, WNV-AUS60 is impaired in its ability to spread beyond the initially infected cells. 

### 2.2. IFN Response to WNV in HDF

**Figure 2:**
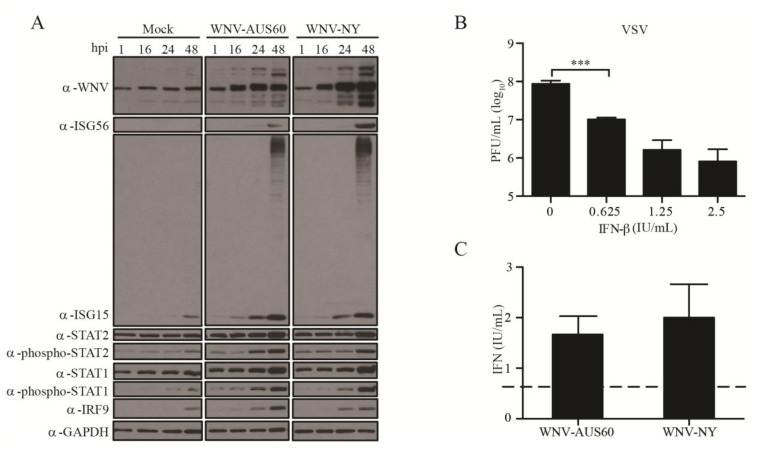
Antiviral response to WNV-AUS60 and WNV-NY in HDFs. (**A**) Steady state protein levels of WNV, ISG56, ISG15, Phospho-STAT-2, STAT-2, Phospho-STAT-1, STAT-1, IRF-9, and GAPDH in mock and WNV-infected (MOI = 0.05) HDF cells. Extracts prepared at the indicated times post-infection were examined by immunoblot. A representative example from three independent experiments is shown; (**B**) Sensitivity of HDFs to IFN. HDF cells were treated with 0, 0.625, 1.25, or 2.5 IU/mL IFN-β for 24 h prior to infection with vesicular stomatitis virus (VSV) (MOI = 1). Supernatants were collected at 24 h post-infection and VSV titers were determined by plaque assay on Vero cells. Values represent the average number of plaque forming units (PFU) per mL (±standard error) from at least three independent experiments. Statistical significance was determined by an unpaired t-test. Asterisks indicate differences that are statistically significant (*** *p* < 0.001); (**C**) Determination of the WNV-induced IFN levels using a VSV-based bioassay on HDFs. HDF cells were treated with specified supernatants for 24 h prior to infection with VSV (MOI = 1). Supernatants were collected at 24 h post-infection and VSV titers were determined by plaque assay on Vero cells. Values represent the level of type-I IFN (IU/mL) (±standard error) from three independent experiments. The dashed line represents the limit of detection for the assay.

The restriction of viral spread within HDF cultures was suggestive of paracrine protection by type-I IFN since WNV infection has previously been shown to be controlled by IFN-α/β in other *in vivo* and *in vitro* models [33,34,35]. Therefore, we measured the levels of type-I IFNs in supernatants recovered from WNV-infected HDFs using a bioassay on A549 cells. While approximately 160 IU/mL of IFN was detected in supernatants recovered from WNV-NY-infected cells at 48 h post-infection, IFN was not detected at 24 h post-infection (data not shown). Moreover, IFN was not detected in supernatants recovered from mock- or WNV-AUS60-infected cells at either 24 or 48 h post-infection (data not shown). Despite the lack of IFN detection, phosphorylated-STAT1 and STAT2 were observed by western blot in lysates recovered from WNV-NY- and WNV-AUS60-infected cultures at 24 h post-infection (Figure 2A). Additionally, both WNV-NY and WNV-AUS60 induced the expression of a panel of interferon stimulated genes (ISGs). Combined, these data suggest that both strains induced IFN responses in HDFs as early as 24 h post-infection, though the level of IFN expression was below the detection limit of 2 IU/mL of a standard bioassay using A549 cells. Therefore, we assessed whether HDFs were sensitive to low levels of IFN. HDFs were treated with 0.625 to 2.5 IU/mL of IFN-β and infected with vesicular stomatitis virus (VSV), a virus that is highly sensitive to the antiviral effects of IFN (Figure 2B). VSV replication in HDFs was suppressed in a dose-dependent manner, indicating that levels as low as 0.625 IU/mL of IFN are capable of inducing an antiviral state within this cell line. 

Based on these results, we reassessed IFN levels in supernatants from WNV-NY and WNV-AUS60 infections using a bioassay on HDFs. Using this more sensitive assay, approximately 2 IU/mL of IFN were detected in supernatants recovered from WNV-NY- and WNV-AUS60-infected cells at 24 h post-infection (Figure 2C). Thus, both viruses induce the expression of low levels of IFN at early times post-infection. 

**Figure 3:**
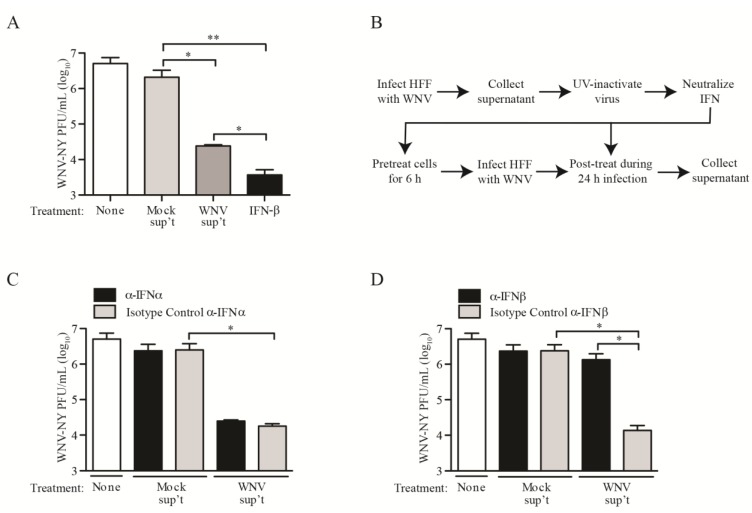
Inhibitory capacity of UV-inactivated supernatants recovered from WNV-AUS60-infected HDFs. (**A**) HDFs were pretreated with UV-inactivated supernatants recovered from mock- or WNV-AUS60-infected cells or 25 IU/mL IFN-β for 6 h prior to infection with WNV-NY (MOI = 0.05). Cultures were incubated for 1 h and the viral inoculum was replaced with UV-inactivated supernatants or 25 IU/mL IFN-β. Supernatants were recovered 24 h post-infection and viral titers determined by plaque assay on Vero cells. Values represent the average number of plaque forming units (PFU) per mL (±standard error) from three independent experiments; (**B**) Schematic of pre- and post-treatment of cells with UV-inactivated supernatants in the presence or absence of neutralizing antibodies to IFN-α or IFN-β; (**C** and **D**) Effects of neutralizing antibodies or specified isotype controls to (**C**) IFN-α or (**D**) IFN-β on the inhibitory capacity of UV-inactivated supernatants recovered from WNV-AUS60-infected HDFs. Values represent the average number of plaque forming units (PFU) per mL (±standard error) from three independent experiments. Statistical significance was determined by an unpaired t-test. Asterisks indicate differences that are statistically significant (* *p* < 0.05 and ** *p* < 0.01).

To confirm that the low levels of IFN produced in response to WNV infection were sufficient to suppress viral replication, we measured WNV-NY infectious particle production in HDFs treated with UV-inactivated supernatants recovered from mock- or WNV-AUS60-infected cultures. WNV-NY titers were reduced by approximately 2 logs in cultures treated with UV-inactivated WNV-AUS60 supernatants compared to cultures treated with mock supernatants (Figure 3A), which was slightly lower than the reduction observed in control cells treated with 25 IU/mL of IFN-β. Similarly, WNV-NY infectious particle production was inhibited in HDFs treated with UV-inactivated supernatants recovered from WNV-NY-infected cultures (data not shown). Moreover, treatment of HDFs with either UV-inactivated WNV-AUS60 or WNV-NY supernatants reduced WNV-AUS60 titers to below detectable levels (data not shown). To confirm that the inhibitory effect of WNV supernatant treatment was due to IFN, the UV-inactivated supernatants were incubated with isotype controls or neutralizing antibodies to IFN-α or IFN-β prior to being used to pre- and post-treat HDFs (Figure 3B). While neutralization of IFN-β had no effect (Figure 3C), addition of IFN-β neutralizing antibodies abrogated the inhibitory effect of the WNV-AUS60 supernatants and restored WNV-NY titers to levels equivalent to those observed in control cells treated with mock supernatants (Figure 3D). Thus, the inhibitory effect of the culture supernatant was due to low levels of secreted IFN-β.

### 2.3. IFN Suppresses WNV Infectious Particle Production at Late Times Post-Infection

**Figure 4:**
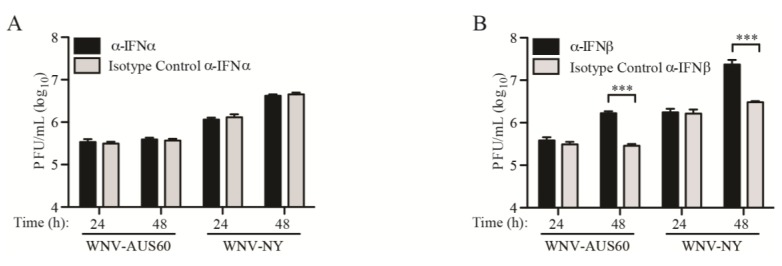
WNV replication in the presence and absence of neutralizing antibodies to IFN-α or IFN-β. HDF cells were infected with WNV-NY or WNV-AUS60 (MOI = 0.05) and the inoculum was removed after 1 h and replaced with complete DMEM containing specified isotype control antisera or neutralizing antibodies to (**A**) IFN-α or (**B**) IFN-β. Culture supernatants were recovered at the indicated times post-infection and viral titers were determined by plaque assay on Vero cells. Values represent the average number of PFU per mL (±standard error) from three independent experiments. An unpaired t-test was performed to determine significance. Asterisks indicate differences that are statistically significant (*** *p* < 0.001).

To directly assess the effect of IFN on WNV replication, we examined infectious particle production in the presence and absence of neutralizing antibodies to IFN-α or IFN-β. As expected, neutralization of IFN-α had no effect on infectious virus production, indicating that IFN-α was not expressed at these time points and therefore, does not play a role in initially controlling WNV replication in human dermal fibroblasts (Figure 4A). Likewise, infectious particle production was unchanged for both WNV-NY and WNV-AUS60 at 24 h post-infection in the presence of neutralizing antibodies to IFN-β and their isotype controls. However, neutralization of IFN-β significantly increased WNV-AUS60 and WNV-NY titers at 48 h post-infection, though WNV-AUS60 titers remained approximately one log lower than WNV-NY (Figure 4B). Thus, IFN-β appears to play a role in controlling WNV infectious particle production at late, but not early, times post-infection. Moreover, this demonstrates that the disparity between WNV-AUS60 and WNV-NY viral set points is regulated by an IFN-independent mechanism.

### 2.4. WNV Strain Variation in Particle Infectivity during HDF Infection

**Figure 5:**
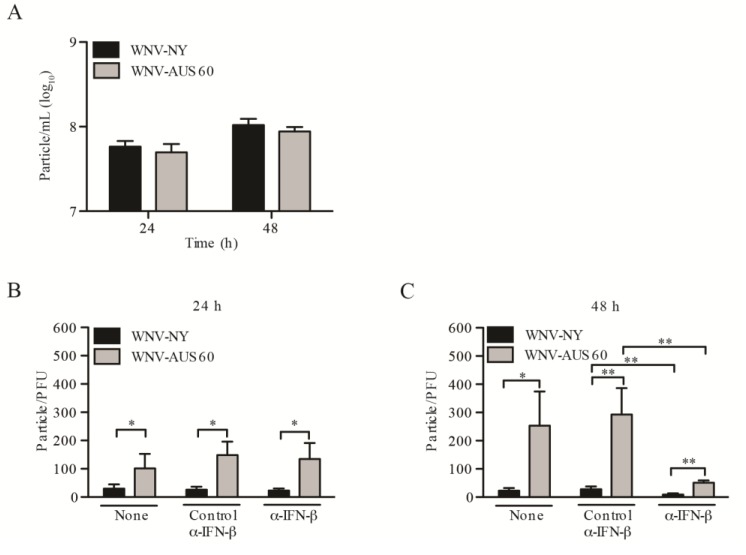
Infectivity of WNV particles produced in HDFs. HDF cells were infected at an MOI of 0.05 with WNV-NY or WNV-AUS60. (**A**) Total viral particle production at 24 and 48 h post-infection was determined using a flow cytometry-based virus counter. Values represent the average number of particles per mL (±standard error) from three independent experiments; (**B**,**C**) WNV infectivity in the presence and absence of specified isotype control or neutralizing antibodies to IFN-β at (**B**) 24 and (**C**) 48 h post-infection. The concentration of total virus particles and infectious particles was determined using a flow cytometry-based virus counter and plaque assays on Vero cells, respectively. Values represent the average particle to PFU ratio of three independent experiments. Statistical significance was determined by an unpaired t-test. Asterisks indicate differences that are statistically significant (** *p* < 0.01 and ** *p* < 0.001).

Key steps in the viral life cycle leading up to infectious particle production include translation and replication of the viral genome, assembly of the virus particle at the ER membrane, transport of virus particles through the secretory pathway, and finally virus release. To assess whether the lower viral set point for WNV-AUS60 was due to impairment at or before the stage of viral assembly and release, we compared WNV-NY and WNV-AUS60 total virus particle production using a virus counter. The virus counter utilizes two dyes in a flow cytometry-based system that simultaneously detects nucleic acid and protein, thereby excluding empty particles and cellular debris from the analysis. Similar levels of total viral particles were detected for WNV-NY and WNV-AUS60 at 24 and 48 h post-infection (Figure 5A), suggesting that WNV-AUS60 and WNV-NY replicate and assemble at a similar rate and to equivalent levels within HDFs. Using the physical counts of total virus particles obtained from the virus counter and the biological counts determined by plaque assay, we determined the particle to PFU ratio for WNV-NY and WNV-AUS60 at 24 and 48 h post-infection (Figure 5B,C). Based on these calculations, the infectivity of WNV-AUS60 particles was significantly reduced at both time points compared to WNV-NY. Notably, yield reduction assays indicated that the reduced infectivity of WNV-AUS60 was not due to the generation of defective interfering (DI) particles (data not shown). These data suggest that the lower viral set point for WNV-AUS60 was due, in part, to WNV-AUS60 particles being less infectious than WNV-NY particles. 

In studies utilizing several different viruses, infected cells treated with IFN produced viral particles with lower infectivity compared to untreated control cells [36,37,38,39,40,41]. To assess whether IFN plays a similar role in reducing WNV infectivity, we examined the effect of IFN-β neutralizing antibodies on the particle to PFU ratios of WNV-NY and WNV-AUS60. Although neutralization of IFN-β had no effect on total particle production (data not shown), the particle to PFU ratios for both WNV-NY and WNV-AUS60 were substantially reduced at 48 h post-infection (Figure 5C); thus, demonstrating that IFN-β plays a role in modulating infectivity of WNV particles late in the course of infection. In contrast, neutralization of IFN-β had no effect on WNV particle to PFU ratios at 24 h post-infection (Figure 5B), which is consistent with our previous data demonstrating that IFN does not regulate viral titers at this time point. Therefore, the higher level of defective particle production observed for WNV-AUS60 at 24 h post-infection was independent of IFN-β and therefore, due to a strain-specific defect in infectious particle production.

## 3. Experimental Section

### 3.1. Cells and Viruses

Primary human dermal fibroblasts (HDFs) derived from neonatal foreskin were kindly provided by Alison McBride (NIH). Vero, A549, and HDF cell lines were propagated at 37 °C in 5% CO_2_ in Dulbecco’s Modified Eagle’s Medium (DMEM) (Mediatech, Manassas, VA, USA) supplemented with 10% fetal bovine serum (FBS) (BioWhittaker, Walkersville, MD, USA), antibiotic/antimycotic solution, and nonessential amino acids (complete DMEM). C6/36 cells were propagated at 28 °C in 5% CO_2_ in Minimal Essential Medium (MEM) (Mediatech) supplemented with 10% fetal bovine serum and antibiotic-antimycotic solution (complete MEM). A WNV-NY strain 3356 stock was generated by passaging virus obtained from the the infectious clone pFL-WNV [32] once on HEK293 cells and twice on C6/36 cells. WNV-AUS60 stock was generated by plaque purifying clinical isolate MRM16, obtained from the World Reference Center of Emerging Viruses and Arboviruses (Galveston, TX, USA), passaging once on HEK293 cells and twice on C6/36 cells. Titers for each stock were determined on Vero and A549 cells using a standard plaque assay. Focus forming assays (see below) were used to determine the titer of each viral stock on HDFs. Vesicular stomatitis virus encoding green fluorescent protein (VSV-GFP) (a gift from Michael A. Whitt) was amplified in BHK-J cells.

### 3.2. Focus-Forming Assays

HDFs were grown in 12-well tissue culture plates and infected with serially-diluted WNV-NY or WNV-AUS60. After 1 h, inoculum was removed and replaced with complete DMEM in 10% methylcellulose. At 24 h post-infection, monolayers were washed three times with phosphate buffered saline (PBS) (Hyclone, Logan, UT, USA) and fixed with 3% paraformaldehyde for 30 min at room temperature. Cell monolayers were permeabilized with a solution of PBS/0.2% Triton X-100, blocked with PBS containing 1% normal goat serum, and incubated with WNV hyperimmune ascitic fluid (1:1000, World Reference Center of Emerging Viruses and Arboviruses) followed by Dylight 549 nm-conjugated goat anti-mouse IgG (1:800, Jackson ImmunoLaboratories, West Grove, PA, USA). Foci were visualized with an Olympus IX51 microscope (Olympus America Inc., Center Valley, PA, USA). 

### 3.3. Virus Growth Curves

Cell cultures were infected with WNV-NY or WNV-AUS60 (MOI = 0.05). The amount of virus added to cultures to achieve the indicated MOI was calculated using the titer of the viral stock as determined on HDFs using a focus forming assay as described above. Cultures were incubated for 1 h at 37 °C with rocking, the inoculum was removed and complete DMEM was added. Culture supernatants were recovered at the indicated times, clarified by low speed centrifugation for 5 min, transferred to new tubes, and stored at −80 °C. Viral titers were determined by plaque assay on Vero cells.

### 3.4. Plaque Assays

Monolayers of Vero cells in six-well plates were inoculated with serial dilutions of viral samples. The cells were incubated in a 5% CO_2_ incubator at 37 °C with rocking for 30 min (VSV) or 1 h (WNV). The inoculum was removed and a 0.9% agarose-complete DMEM overlay added. VSV plaques were counted 24 h post-infection. For WNV titration, cell monolayers were incubated for 48 h and a second overlay of agarose-containing complete DMEM supplemented with 0.003% neutral red (ICN Biomedical, Irvine, CA, USA) was added. The plates were incubated for an additional 48 (WNV-NY) to 72 h (WNV-AUS60) prior to counting plaques. All titers were performed in duplicate.

### 3.5. Quantitative Real-Time PCR

Total RNA was extracted from HDFs infected with WNV (MOI = 0.05) using TRIzol reagent (Invitrogen Life Technologies, Inc., Grand Island, NY, USA) and treated with Turbo DNA-free (Invitrogen). RNA levels were determined by quantitative real-time PCR (qRT-PCR) analysis on a Roche LC480 using Veriquest One-Step SYBR green MasterMix (Affymetrix Biosystems, Santa Clara, CA, USA) with 25 ng of RNA. The following primers were used: WNV(s): 5′ TGGAACCACCCTTTGGAG 3′; WNV(as): 5′ GTCCCAAGCTGTGTCTCC 3′; hGAPDH(s): 5′ CCACTCCTCCACCTTTGAC 3′; hGAPDH(as): 5′ ACCCTGTTGCTGTAGCCA 3′. 

### 3.6. Immunofluorescence Assay (IFA)

HDFs were grown on coverslips and infected with WNV-AUS60 or WNV-NY at an MOI of 0.05. After 1 h, inoculum was removed and replaced with complete DMEM. At 24 h post-infection, monolayers were washed with PBS and fixed with 3% paraformaldehyde for 30 min at room temperature. Cell monolayers were permeabilized with a solution of PBS/0.2% Triton X-100, blocked with PBS containing 1% normal goat serum, and incubated with WNV hyperimmune ascitic fluid (1:1000, World Reference Center of Emerging Viruses and Arboviruses) followed by Dylight 549 nm-conjugated goat anti-mouse IgG (1:800, Jackson ImmunoLaboratories) and Hoescht stain (0.1 µg/mL). Cells were visualized with an Olympus IX51 microscope equipped with a digital camera. 

### 3.7. Flow Cytometry

Cultures of HDFs grown on 6-well plates were infected with WNV at an MOI of 0.05. At the indicated times post-infection, cells were removed from plates by trypsinization, washed twice with PBS and fixed in 3% paraformaldehyde. Cells were permeabilized with PBS/0.2% Triton X-100, blocked in PBS containing 0.5% heat-inactivated FBS and probed with WNV hyperimmune ascitic fluid (1:1000, World Reference Center of Emerging Viruses and Arboviruses) followed by DyLight 549 nm conjugated goat anti-mouse IgG (1:2000). For flow cytometry analysis, 100,000 single cell events were collected using a FACS Canto (BD Biosciences, San Jose, CA, USA).

### 3.8. Immunoblot Analysis

Cells were washed twice with PBS and lysed in RIPA buffer (10 mM Tris, 150 mM NaCl, 0.02% Na-deoxycholate, 1% Triton X-100, 0.1% sodium dodecyl sulfate [SDS]) containing protease inhibitors (Sigma-Aldrich, St. Louis, MO, USA). Proteins (20 µg) were resolved on 10%–12% polyacrylamide gels containing SDS and transferred to NitroPure nitrocellulose membranes (Micron Separations Inc., Westborough, MA, USA). Blots were blocked overnight at 4 °C and probed with the following monoclonal or polyclonal antibodies: polyclonal rabbit anti-GAPDH (1:4000; Abcam, Cambridge, MA, USA), polyclonal mouse anti-WNV hyperimmune ascetic fluid (1:1000; World Reference Center of Emerging Viruses and Arboviruses), polyclonal rabbit anti-ISG56 (1:2000; kindly provided by Dr. Ganes Sen), polyclonal rabbit anti-ISG15 (1:2500; kindly provided by Dr. Arthur Haas), polyclonal rabbit anti-phospho-STAT-2 (1:1000; Millipore, Billerica, MA, USA), polyclonal rabbit anti-STAT-2 (1:1000; Santa Cruz, Dallas, TX, USA), monoclonal mouse anti-phospho-STAT-1 (1:100; Santa Cruz), polyclonal mouse-anti-STAT-1 (1:1000; Cell signaling, Boston, MA, USA), and polyclonal rabbit anti-IRF-9 (1:200; Santa Cruz). Following a secondary incubation with peroxidase-conjugated goat anti-rabbit or goat anti-mouse (Millipore) and treatment with ECL+ Western Blotting detection reagents (Amersham Biosciences, Piscataway, NJ, USA), the protein bands were visualized by exposure of the membrane to film.

### 3.9. UV-Inactivation

UV-inactivation was carried out by exposing a 1 mL aliquot of supernatant recovered form mock- or WNV-infected HDF to UV (254 nm) for 2 min at room temperature in a Stratalinker Model XL-1000 (Spectronics Corp., Westbury, NY, USA). Titers of UV-treated supernatants were below detectible levels by plaque assay on Vero cells, confirming complete inactivation of the WNV-infected supernatants. 

### 3.10. Interferon Bioassay

A549 or HDF cells in 24-well plates were treated with two-fold serial dilutions of human IFN-β (BEI Resources, Manassas, VA, USA) or cell-free, UV-inactivated supernatants recovered from mock or WNV-infected HDFs. Cultures were incubated for 24 h at 37 °C, infected with VSV (MOI = 1) and supernatants were collected at 24 h post-infection. VSV titers were determined by plaque assay on Vero cells as described above. IFN concentrations were determined based on a standard curve generated from the titers of VSV recovered from samples treated with serial dilutions of IFN-β.

### 3.11. Neutralization of Type-I IFN

The antibody concentration necessary to neutralize the IFN present in supernatants recovered from WNV-infected HDFs was determined by pretreating A549 cells for 24 h with 25 IU/mL of IFN-α or IFN-β in the presence of 2-fold serial dilutions of the antibodies to IFN-α (NR-3089; BEI resources) or IFN-β (NR-3091; BEI resources). Control wells consisted of cells treated with IFN only, no IFN or isotype matched antisera to IFN-α or IFN-β. Pretreated cells were infected with VSV (MOI = 1) and supernatants were collected at 24 h post-infection. Viral titers were determined by plaque assay on Vero cells. Culture supernatants were neutralized with twice the amount of antibody necessary to neutralize 25 IU/mL IFN-α or IFN-β or the appropriate control antisera (NR-3089 or NR-3090; BEI resources) for 1 h at 37 °C. For neutralization during WNV-infection, HDFs were inoculated with WNV (MOI = 0.05) for 1 hour at 37 °C and the inoculum was replaced with complete DMEM containing neutralizing antibodies to IFN-α/IFN-β or the appropriate control antisera. In wells that contained supernatants to be collected at 48 h post-infection, supplemental antisera were added at 24 h post-infection. 

### 3.12. Detection and Enumeration of Total Virus Particles

Culture supernatants were cleared by low speed centrifugation for 5 min and analyzed using the Virus Counter 2100 (ViroCyt LLC, Denver, CO, USA) as per the manufacturer’s instructions. Briefly, samples were diluted 1:10 or 1:30 to a total volume of 100 µL and incubated in the dark for 30 min with 50 µL of Combo dye, which stains nucleic acid and protein. Two-channel fluorescence was used to detect co-localization of nucleic acid and protein. Events with simultaneous detection within both channels were defined as virus particles by Virocyt software.

### 3.13. Statistical Analysis

Graphpad Prism 5 (GraphPad Software, Inc., La Jolla, CA, USA) was used for all statistical analyses. Comparative significance was determined with unpaired Student’s t-tests.

## 4. Conclusions

Here we demonstrate that both nonneuroinvasive and neuroinvasive strains of WNV are capable of establishing an infection within human dermal fibroblasts. However, a strain-specific defect in infectious particle production limits the ability of WNV-AUS60 to disseminate from the initial site of infection, resulting in a lower viral set point compared to WNV-NY. The defect in WNV-AUS60 infectious particle production was not specific to human dermal fibroblasts. WNV-AUS60 exhibited both a smaller plaque phenotype and reduced titers compared to WNV-NY on Vero cells (data not shown). It is unclear why WNV-AUS60 particles are less infectious than those of WNV-NY. A recent study demonstrated that mutant alphaviruses with higher error rates are reduced in their infectivity compared to wild-type [42]. Therefore, one possible explanation for this deficiency is that the RNA dependent RNA polymerase (RdRp) encoded by WNV-AUS60 has an increased error rate that pushes this strain over the error threshold, thus, resulting in an increased production of defective particles. Alternatively, the lower infectivity of WNV-AUS60 particles may be due to a reduced capacity to undergo maturation. *Flavivirus* particles are secreted from the infected cell as a combination of mature, partially mature and immature virions. Recent reports have demonstrated that partially mature viral particles are still capable of infecting cells, however, immature *Flavivirus* particles are noninfectious due to an inability to efficiently bind to the target cell [43,44,45]. Therefore, an increase in the percentage of immature particles released from the infected cell would result in decreased infectivity of WNV-AUS60 particles. 

We also demonstrated that WNV replication is restricted late in infection through an IFN-β-mediated reduction in viral infectivity. While many strains of WNV are capable of inhibiting IFN signaling [46,47,48,49,50,51,52,53,54,55], ISG expression is not completely suppressed during WNV infection [34,53,56,57,58,59]. Recent studies have defined the mechanisms by which several of these antiviral proteins suppress WNV infectious particle production [60,61]. However, none of the antiviral proteins characterized to date decrease particle infectivity. Therefore, the antiviral effector protein(s) responsible for the IFN-β-dependent decrease in particle infectivity we observed remains to be determined. Conceivably, this reduction in viral infectivity may limit the ability of the dermal layer to serve as a productive reservoir for WNV infection. Moreover, the higher level of defective particles that are produced late in infection may help to stimulate the adaptive immune response to WNV. Since the infectivity of WNV-AUS60 is inherently lower than WNV-NY, the IFN-dependent decrease in infectious particle production may have a greater impact on the ability of this strain to disseminate from the site of infection and ultimately cause disease.
